# Early initiation of breastfeeding: a systematic literature review of factors and barriers in South Asia

**DOI:** 10.1186/s13006-016-0076-7

**Published:** 2016-06-18

**Authors:** Indu K. Sharma, Abbey Byrne

**Affiliations:** Nossal Institute for Global Health, The University of Melbourne, Melbourne, VIC Australia

**Keywords:** Breastfeeding, Barriers, Factors, Early initiation of breastfeeding, Timely initiation of breastfeeding, South Asia, Breastfeeding within 1 h of birth, Colostrum, Review

## Abstract

**Background:**

Early or timely initiation of breastfeeding is crucial in preventing newborn deaths and influences childhood nutrition however remains low in South Asia and the factors and barriers warrant greater consideration for improved action. This review synthesises the evidence on factors and barriers to initiation of breastfeeding within 1 h of birth in South Asia encompassing Afghanistan, Bangladesh, Bhutan, India, Maldives, Nepal, Pakistan and Sri Lanka.

**Methods:**

Studies published between 1990 and 2013 were systematically reviewed through identification in Academic Search Complete, CINAHL, Global Health, MEDLINE and Scopus databases. Twenty-five studies meeting inclusion criteria were included for review. Structured thematic analysis based on leading frameworks was undertaken to understand factors and barriers.

**Results:**

Factors at geographical, socioeconomic, individual, and health-specific levels, such as residence, education, occupation, income, mother’s age and newborn’s gender, and ill health of mother and newborn at delivery, affect early or timely breastfeeding initiation in South Asia. Reported barriers impact through influence on acceptability by traditional feeding practices, priests’ advice, prelacteal feeding and discarding colostrum, mother-in-law’s opinion; availability and accessibility through lack of information, low access to media and health services, and misperception, support and milk insufficiency, involvement of mothers in decision making.

**Conclusions:**

Whilst some barriers manifest similarly across the region some factors are context-specific thus tailored interventions are imperative. Initiatives halting factors and directed towards contextual barriers are required for greater impact on newborn survival and improved nutrition in the South Asia region.

**Electronic supplementary material:**

The online version of this article (doi:10.1186/s13006-016-0076-7) contains supplementary material, which is available to authorized users.

## Background

Child survival is an ongoing public health priority in the South Asia region, which includes eight countries - Afghanistan, Bangladesh, Bhutan, India, Maldives, Nepal, Pakistan and Sri-Lanka [1]. Countries within the region have made significant progress towards Millennium Development Goal 4 (MDG 4) to reduce the under-five mortality rate (U5MR) by two-thirds by 2015 [2]. However the regional U5MR remains off target at 58 deaths per 1000 live births compared to the target of 42 deaths per 1000 live births [3]. Progress has largely been for children aged 1–59 months and now the critical priority is mortality among newborns (birth to 28 days). In the South Asia region, the neonatal mortality rate (NMR) now accounts for 53 % of the U5MR, in contrast to 34 % in Sub-Sahara Africa, and comprises 40 % of all newborn deaths in developing regions of the world [3]. The burden is unequal within the region: the NMR (expressed as deaths per 1000 live births) in 2012 was estimated to be as high as 42 in Pakistan yet as low as six in Sri-Lanka and the Maldives [3]. Improving newborn survival is critical for further reductions in U5MR and achievement of MDG 4, particularly in the South Asia region.

Early or timely initiation of breastfeeding, specifically within 1 h of birth, refers to the best practice recommendation by the World Health Organization (WHO) [4]. A recent systematic review and meta-analysis revealed that breastfeeding initiation after the first hour of birth doubles the risk of neonatal mortality [5]. In specific countries, initiating within 1 h reduced deaths by 19 % in Nepal [6] and 22 % in Ghana [7]. The evidence, drawn from meta-analysis and over 63 developing countries, shows that early initiation of breastfeeding prevents newborn infections, averts newborn death due to sepsis, pneumonia, diarrhoea and hypothermia, and facilitates sustained breastfeeding [8]. In South Asia, merely 41 % of newborns are breastfed within 1 h of birth [1]. Several South Asian countries have some of the worst early initiation of breastfeeding practices in the world; the rates in Pakistan, India, Bangladesh and Nepal are only 29, 41, 47 and 45 % respectively [1].

Insufficient attention is afforded to the public health issue of early or timely initiation of breastfeeding, and the causes of poor practice, even though this preventive intervention is highly cost-effective [6, 7, 9]. Breastfeeding research predominantly focuses on exclusive breastfeeding to the age of 6 months and other infant and young child feeding (IYCF) indicators [7, 10, 11]. Existing systematic literature reviews on early initiation primarily draw on evidence from developed countries and on the effect of skin-to-skin contact on breastfeeding rates [12–15]. It is important to understand the factors associated with delayed breastfeeding initiation and the existing barriers and facilitators to early initiation in order to design and deliver effective strategies to improve the practice [7] and accelerate progress in newborn survival.

This systematic literature review synthesises published evidence on the factors and barriers associated with the initiation of breastfeeding within 1 h of birth in South Asian countries to inform a future of relevant, context-specific actions.

## Methods

Protocol of the systematic literature review was proposed in the University of Melbourne Master of Public Health research project and was approved by the University prior to commencement. The search was undertaken from July to September, 2013. The methods and reporting were developed and conducted with systematic methodology and consistent with the PRISMA reporting guidelines [16].

### Source of literature

This drew on published literature in the electronic bibliographic databases of: Academic Search Complete, Cumulative Index to Nursing and Allied Health (CINAHL), Global Health, MEDLINE Web of Knowledge and Scopus and supplemented by scanning the reference lists of papers included for review.

### Search terms

Search terms were applied with various Boolean operators for three core concepts: breastfeeding; timing of breastfeeding initiation; and countries in South Asia.

The various search terms are:

Breastfeed* or “Breast feed*” or Breastfed* or Breast-fed or “Breast fed*” Breast-feed* or “breast milk” or “breastmilk” or “breast-milk”

Initiat* or colostrum or “pre-lacteal” or “pre lacteal” or prelacteal or “early” or delay South Asia*" or “South East Asia*” or “Southern Asia*” or “South Eastern Asia*” or “Southern East Asia*” or SEAR or Afghan* or Bangladesh* or Bhutan* or India* or Maldives or Nepal* or Pakistan* or “Sri Lanka*”.

MeSH heading was used for breastfeeding; and ASIA South-eastern or Asia. A detailed search strategy of one database is presented in Additional file 1.

### Inclusion and exclusion criteria

The eligibility of studies for review was assessed on a set of four inclusion and exclusion criteria, based on the reporting of factors and/or barriers, timing of breastfeeding initiation, country, year, language, study design and full text availability. The set of criteria are shown in Table 1. Identification of barriers was based on ‘*issues that drive the reasons why people do not have or make use of services’*, as the widely applied definition in literature and by Jacobs et al. (2011) [17] in the analytical framework for analysis of health service barriers.

**Table 1:** Inclusion and exclusion criteria

Inclusion criteria	Exclusion criteria
Countries in South Asia encompassing 8 countries namely Afghanistan, Bangladesh, Bhutan, India, Maldives, Nepal, Pakistan and Sri Lanka.	Studies conducted among South Asian women living in other regions
Studies published in or after 1990. In English language	Full-text not accessible
Published quantitative, qualitative and mixed method studies	Studies not demonstrating a clear research methodology - commentaries, letters and editorials.
Studies reporting factors or barriers on initiation of breastfeeding within 1 h of birth.	Studies on initiation of breastfeeding after 1 h of birth.

### Study selection and data extraction

Studies retrieved from databases were exported to Endnote X5 and duplicated citations were removed. Abstracts were screened for relevance to the study question and country of the study. All other inclusion and exclusion criteria were applied through assessment of the full text publications.

Studies selected for inclusion were transferred to a Microsoft Excel spreadsheet for extraction of data items of: setting, population, methods, factors and reported barriers to early initiation of breastfeeding, and for thematic analysis.

### Quality appraisal

Quality of included studies was appraised separately for qualitative and quantitative methods assessing features of study design, methodology and analysis. Studies were classified into strong, moderate and weak based on criteria set within two different tools: Critical Appraisal Skills Programme (CASP) and Effective Public Health Practice Project (EPHPP) tools. Qualitative studies were appraised using the CASP tool which contains a checklist of ten screening questions regarding the aim of the research, appropriateness of the qualitative methodology, appropriateness of research design to address aim, appropriateness of recruitment strategy, data collection methods, relationship between researcher and participants, ethical issues, data analysis, statement of findings and value of research [18]. This tool has previously been evaluated, revised and reviewed [19]. Quantitative studies were appraised using the EPHPP tool to rate studies based on given criteria on the basis of: selection bias, study design, confounders, blinding, data collection methods, withdrawals and drop-outs, intervention integrity (for intervention studies) and analyses [20, 21]. This tool has demonstrated high inter-reliability across individual domains and high intra-class correlation coefficient value [20]. For mixed-method studies, the CASP tool was applied to the qualitative elements and the EPHPP tool to the quantitative elements.

These quality appraisal methods and associated rankings assess the validity of individual studies. These are not a means to weighting the magnitude of study findings between studies, rather the conclusions of the quality appraisal indicate the rigor of the study and through such the confidence, or weight, with which the study findings can be taken.

### Synthesis of results

The results were synthesized according to the two features being addressed; the factors, and the barriers, associated with delayed initiation of breastfeeding. The results concerning factors were synthesized systematically according to the level at which the factors exert influence on early breastfeeding initiation. This approach was based on the framework for analysis of barriers with a health system lens established by The SURE Collaboration for structured and systematic analyses [22]. The levels relevant to the factors of this health issue were identified to be: geographical, health-specific, socio-economic, and individual. The results on barriers were synthesised using thematic analysis and arranged based on the analytical framework of barriers affecting health care in low-resource Asia settings developed through a review by Jacobs et al. [17], adapted from Peters et al. [23] and Ensor and Cooper [24]. This analytical framework provided a structured and comprehensive perspective on barriers experienced in the health sector, categorised as accessibility, availability and acceptability barriers in terms of both supply and demand [17].

## Results

### Study selection

**Fig. 1:**
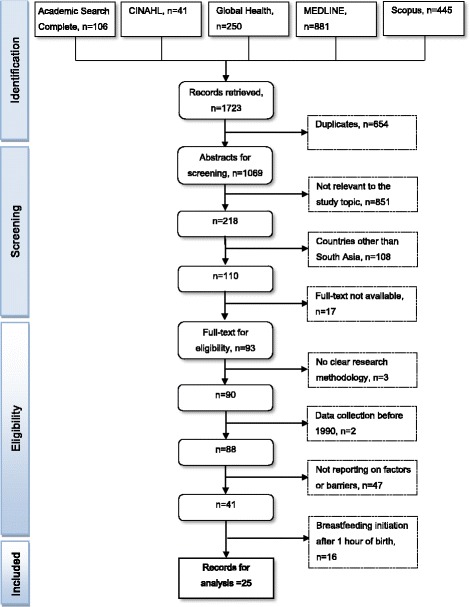
Flow chart of selection process

The search strategy retrieved 1723 studies. After applying the process of selection, summarised in Fig. 1, 25 studies were included for review. Scanning reference lists of reviewed articles did not produce additional results, suggesting that the search was comprehensive.

Studies selected for review represented Bangladesh (four), India (eight), Maldives (one), Nepal (three), Pakistan (six) and Sri Lanka (two). One study was multi-country across Bangladesh, India, Nepal and Sri Lanka while no studies were retrieved from Afghanistan and Bhutan. Two studies used qualitative methods, 17 used quantitative methods and six were mixed-methods studies.

Participants in the included studies were ever-married women of reproductive age, typically with at least one child; traditional birth attendants (TBAs); mother-in-laws; and fathers. Several studies involve random selection of participants while others targeted new mothers and fathers, untrained TBAs, ethnic minority women, attendees of immunisation clinics, postnatal mothers, mothers who were currently breastfeeding and those who had discontinued breastfeeding.

The summarised characteristics of included studies are presented in Table 2, with study details in Additional file 2.

**Table 2:** Summary of included studies

Serial number	Source	Study setting	Outcomes – reported factors or barriers	Study methods	Quality grading
1	Dibley et al., 2010 [43]	Bangladesh, India, Nepal and Sri Lanka	Delivery by caesarean section Home delivery No antenatal check-up No decision-making participation Lack of access to media	Quantitative cross sectional survey: secondary analysis of DHS	Moderate
2	Haider et al., 2010 [41]	Dhaka, Bangladesh	Lack of knowledge No milk secretion Traditional belief Mother’s ill-health Baby’s ill-health Grandmothers’ decision Midwife discouraged Lack of support	Quantitative cross-sectional survey. Qualitative	Weak quantitative and moderate qualitative
3	Parveen et al., 2012 [39]	Haryana, India	Custom Mother’s illness No milk secretion	Quantitative cross-sectional survey	Weak
4	Kaushal et al., 2005 [44]	Haryana, India	Custom	Quantitative cross-sectional survey. Qualitative	Weak quantitative and qualitative
5	Badruddin et al., 1997 [37]	Karachi, Pakistan	Mother unable to sit Caesarean section delivery Night time High BP of mother	Quantitative: longitudinal design. Qualitative	Weak quantitative and moderate qualitative
6	Dihidar et al., 2002 [33]	Calcutta, India	Living in rural area	Quantitative cross sectional survey	Weak
7	Senarath et al., 2012 [25]	Sri Lanka	Male child Low birth weight Home delivery Caesarean section delivery Rural Geography: North Central Mother’s age15-19 years Birth order; first birth No previous birth Living in Sabaragamuwa	Quantitative cross-sectional survey: secondary analysis of DHS 2006-07	Moderate
8	Mihrshahi et al., 2010 [29]	Bangladesh	No maternal education No education of husband Birth order > 5 Home delivery No antenatal check-ups Mothers not watching television Poorest household No decision-making participation Geography: lowest in Barisal	Quantitative cross-sectional survey: secondary analysis of DHS 2004	Moderate
9	Pandey et al., 2010 [28]	Nepal	Caesarean section delivery Delivery assistance from health professionals compared to TBAs No participation in decision making Living in mountainous region	Quantitative cross-sectional survey: secondary analysis of DHS 2006	Moderate
10	Seranath et al., 2010 [35]	Sri Lanka	Birth order; first birth Caesarean section delivery No antenatal visits by midwife Geography: Colombo feeder area	Quantitative cross-sectional survey: secondary analysis of DHS 2000	Moderate
11	Hazir et al., 2013 [27]	Pakistan	Working mothers Caesarean section delivery Residing in Sindh Province	Quantitative cross-sectional survey: secondary analysis of DHS 2006/07	Moderate
12	Khadduri et al., 2008 [47]	Haripur district, Pakistan	Custom; tradition of prelacteal feeding	Qualitative	Moderate
13	Bandyopdahyay et al., 2009 [48]	Rural Bengal, India	Customs; perception that first milk is harmful to the baby; insufficient milk; that milk will only come after 48 h	Qualitative	Weak quantitative and moderate qualitative
14	Patel et al. 2010 [26]	India	No education Mothers aged 15–19 years No education of husband Home delivery Caesarean section delivery No antenatal check-ups Bivariate analysis No post natal check-ups Lowest wealth quintile No participation in decision making No media – radio, newspaper, television Geography: rural area; Central region	Quantitative cross-sectional survey: secondary analysis of National Family Health Survey 2005-06	Moderate
15	Subedi et al. 2012 [31]	Chepang community, Nepal	Illiterate No antenatal check-ups Home delivery	Quantitative cross- sectional survey	Weak
16	Subba et al. 2007 [34]	Pokhara, Nepal	Nuclear family Smaller family size	Quantitative cross-sectional survey	Weak
17	Abdulraheem and Binns 2007 [42]	Maldives (several islands)	Caesarean section delivery	Quantitative cross-sectional survey	Weak
18	Athavale et al. 2004 [36]	Urban Health Centre, Nagpur, India	Caesarean section delivery Customs; prelacteal feeding, discarding colostrum Premature baby Birth order; first birth	Quantitative cross-sectional survey	Weak
19	Ekambaram et al. 2010 [38]	Tertiary care hospital, South India	Child was sick (34 %) Delay in shifting from labour room (25 %) Mother’s motivation/too tired: no consciousness (14 %) Baby was sleeping (5 %)	Quantitative cross-sectional survey	Weak
20	Moran et al. 2009 [49]	Dhaka, Bangladesh	Perceptions of no milk supply	Quantitative cross-sectional survey. Qualitative	Weak quantitative and moderate qualitative
21	Fikree et al. 2005 [46]	Karachi, Pakistan	Customs; traditional feeding practices and perceived health benefits	Quantitative cross-sectional survey. Qualitative	Moderate quantitative and moderate qualitative
22	Rahman et al. 2011 [30]	Bangladesh	No antenatal check-ups Poorest wealth quintile Delivery assistance by non-medically trained provider No Education	Quantitative cross-sectional survey: analysis of Demographic and Health Survey 2007	Moderate
23	Ali et al. 2011 [32]	Semi-urban Pakistan	Lack of education	Quantitative cross-sectional survey	Weak
24	Digra et al. 2012 [45]	Jammu, India	Self-decision (22.2 %) Advice of priest (35 %) Advice of elderly lady in family (20.4 %)	Quantitative cross-sectional survey	Weak
25	Premani et al. 2011 [40]	Karachi, Pakistan	Mothers too tired after delivery	Qualitative	Weak

### Quality of studies

Based on the CASP criteria, both qualitative studies reviewed were of moderate quality owing to limitations in the research design, recruitment strategy and data analysis. Based on the EPHPP, none of the quantitative studies were high-quality ranking because all were moderately-weighted cross-sectional design. Eight studies were moderate quality, while nine were weak based on design, unreliable data collection method and no controlling for confounding factors. Of the six mixed-method studies, four were weak in quantitative and moderate in qualitative design; one was moderate and one was weak in both qualitative and quantitative design.

### Factors associated with early initiation of breastfeeding

**Fig. 2:**
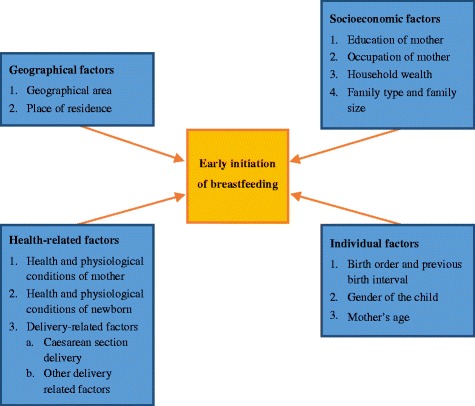
Factors associated with early initiation of breastfeeding

The factors associated with timely or early initiation of breastfeeding as revealed by the existing literature, according to the levels for analysis, are: geographical, socioeconomic, individual and health-specific. The results pertaining to factors are detailed below, and presented in summary in Fig. 2.

#### Geographical factors

Geographical factors found to have a pattern with early initiation of breastfeeding varied across countries. Delayed initiation rate is higher for those living in North Central Province in Sri Lanka [25], Central region in India [26], Sindh province in Pakistan [27] and lower in the plains (Tarai) region of Nepal [28]. Although residing in a rural area was associated with delayed initiation in India [26] and Sri Lanka [25], residing in urban areas was significantly associated with delayed initiation in Bangladesh [29]. All five of these studies have moderate quality grading.

#### Socioeconomic factors

The social and economic circumstances of a woman and the household have much influence on timing of breastfeeding initiation in the South Asian context, pertinently the education of mother, occupation of mother, household wealth and family size and family type. Delayed initiation of breastfeeding is more prevalent among women who have no formal education in Bangladesh [29, 30], India [26], Nepal [31] and Pakistan [32]. In Bangladesh, delayed initiation is associated with low schooled husbands [29]. However, working status of mothers is contrasting depending on the setting. Working mothers in Pakistan are more likely to delay initiation compared to non-working mothers [27] whilst in India non-working mothers are more likely to delay initiation [26]. Breastfeeding initiation based on wealth also contrasts between countries. Delayed initiation is more likely by women from poorest households in Bangladesh [29, 30] yet by those of the richest households in Sri Lanka [25]. In an urban area of India, Kolkata, early initiation practices were higher among women from lower-income groups [33]. In terms of family type and size, women with nuclear families (not residing with the mother-in-law), with fewer children, are more likely to delay initiation in Nepal [34]. Results relating to socioeconomic influence from studies in India and Bangladesh [25–27, 29, 30] are more strongly founded based of moderate quality grading, whilst those of Nepal, Pakistan and Sri Lanka [31–34] were of weak quality grading.

#### Individual factors

Birth order, previous birth interval, teenage motherhood and having a male child are linked with early initiation of breastfeeding. For their first-born child, women are less likely to initiate breastfeeding within 1 h of birth, as reported in Sri Lanka [25, 35], India [36] and Pakistan (no association in multivariate analysis) [27]. Additionally, delayed initiation is more likely for children of five or later birth order in Bangladesh [29]. Similarly, children of teenage mothers (aged 15–19 years) and male child were less likely to be breastfed within 1 h of birth [25]. Most of the studies reporting the individual factors of timely or early initiation of breastfeeding are with moderate quality except one high quality [25].

#### Health related factors

Many reviewed studies specify health and physiological conditions of mother, health and physiological condition of newborn and delivery factors as health related factors associated with either the practice or non-practice of early initiation of breastfeeding.

Five studies reported the mother’s health condition as a reason for delaying breastfeeding [37–41], specifically being unconscious after delivery [38, 41], unable to sit, experiencing hypertension [37], fatigue [40], or generalised ‘illness’ after delivery [39].

Of the newborn, early initiation is comparatively lower among children of low birth weight [25], prematurity [36], and being ill or considered weak [38, 41].

Delivery-related conditions have also been identified as a factor in the practice of early initiation of breastfeeding in South Asia. Seven qualitative studies conducted in Bangladesh [41], India [26, 36], Nepal [28], Pakistan [27], Maldives [42] and Sri Lanka [35] highlighted delivery by caesarean section as a major factor. Similarly, two other studies specified that time for recovery from caesarean delivery [37] and delay in uniting the newborn and mother after caesarean section [41] as reasons for delayed initiation. Moreover, three studies reported specific care practices as factors to early initiation of breastfeeding among facility-based births, namely late delivery of the placenta [41], allocated time for recovery from delivery [37], delay in shifting women from the labour room [38], and delivery during the night [37].

### Barriers to early initiation of breastfeeding

The identified barriers to the early initiation of breastfeeding in South Asia have been synthesised as supply side and demand side barriers in terms of accessibility, availability and acceptability, as presented in Table 3.

**Table 3:** Barriers to early initiation of breastfeeding

Supply-side barriers	Demand-side barriers
Acceptability	Acceptability 1. Traditional feeding practices 1.1 breastfeeding according to time of birth 1.2 bathing rituals 1.3 prelacteal feeding and discarding colostrum 2. Advice of priests 3. Influence of mother in law
Availability 1. Lack of knowledge and misperception	Availability 1. Lack of support 2. Milk insufficiency
Accessibility 1. No or few antenatal appointments 2. Home delivery 3. Type of delivery assistance and practices 4. No post-natal check-up	Accessibility 1. Low socio economic status linked to lack of access to media: radio and newspaper 2. Mother’s involvement in decision making

#### Supply-side barriers

##### Barriers to availability

Lack of availability of information for correct knowledge and misperception on breastfeeding was reported as a barrier. Lack of knowledge on the importance of early initiation and the perception that water must be given to the newborn because breast milk alone will not sustain the baby were observed in Bangladesh [41]. However, for the quantitative data the study based findings only on descriptive values without statistical associations.

##### Barriers to accessibility

Nine studies, eight of which were moderate quality grading [25, 26, 28–30, 35, 41, 43], reported barriers to accessing initiation of breastfeeding in terms of antenatal and postnatal check-up, home delivery, and delivery by non-skilled attendants. No or few antenatal appointments, home delivery, delivery assistance and practices and no post-natal check-up have been reported in literature as supply side barriers to accessibility in terms of facilitation of breastfeeding practice. Six studies consistently reported no or few antenatal appointments as a barrier to early initiation of breastfeeding in Bangladesh [29, 30], India [26], Nepal [31] and Sri Lanka [35, 43]. In terms of delivery, home delivery is linked with delayed initiation, shown in Bangladesh [29], India [26, 43], Sri Lanka [25] and Nepal [31]. Similarly, early initiation of breastfeeding is lower for women assisted by TBAs or friends/relatives during delivery in India compared to health professionals [26]. In contrast, in Nepal women assisted by TBAs are less likely to delay initiation compared to those assisted by health professionals [28]. In Bangladesh study results are conflicting, with one study reporting early initiation with birth assistance by medically trained providers [30], yet in a qualitative study mothers described that midwives discourage breastfeeding for first 3 days [41]. Moreover, women not receiving a postnatal check-up from a public health midwife are more likely to delay breastfeeding initiation compared to those receiving postnatal check-up in Sri Lanka [35], and India [26].

#### Demand-side barriers

##### Barriers to acceptability

Four weak [36, 39, 44, 45] and four moderate quality studies [41, 46–48] highlight traditional feeding practices as demand side barriers to acceptance of early initiation of breastfeeding in South Asia. Specifically, breastfeeding according to time of birth and advice of priest, use of prelacteal feeds and discarding colostrum, and influence of mother in law are observed. A study conducted in Haryana of India revealed the practice of initiating breastfeeding in the evening after seeing stars if the child was born in morning and if the birth was in the night breastfeeding was started within a few hours or early morning [44]. In Bangladesh bathing rituals for mother and newborn must take place before initiating breastfeeding [41]. Moreover, a study conducted in Jammu of Kashmir State revealed advice of priests as a reason for delayed initiation of breastfeeding [45]. Negative perception of colostrum and the use of prelacteal feeds are common barriers, shown in four studies. In Pakistan women reported discarding colostrum, withholding breastfeeding and replacing with prelacteal feeding which is typically administered via a finger of an elderly person and perceived to clean the stomach and strengthen the newborn [46]. Another study described the perception that colostrum may harm or even kill the newborn because it is dirty and stored for 9 months in the breast [47]. Likewise, in a rural area of India mothers perceive that the first milk is harmful to the baby [48]. Mothers in urban India who accept giving colostrum are more likely to initiate breastfeeding within 1 h of the birth [36]. Influence of mother in law and/or elder women has also been observed as barrier, with decision-making around maternal and newborn care reportedly as a role of elderly women of family in India [39], and mother-in-law in India [45], Bangladesh [41], and Pakistan [46].

##### Barriers to availability

Lack of available support and milk insufficiency are demand side barriers. A study from Bangladesh reported lack of support as a barrier to early initiation of breastfeeding [41]. Milk insufficiency is reported by four studies (of weak to moderate quality grading) as the reason for not initiating breastfeeding within 1 h of birth [39, 41, 48, 49].

##### Barriers to accessibility

Our review highlights two major types of barriers to access to information regarding the initiation of breastfeeding. Firstly, two moderate quality studies reveal lack of access to media, linked with low socio-economic status of a household and area, as reported barriers to early initiation of breastfeeding in South Asia. Women not watching television in Bangladesh [29] and India [26], and mothers not listening to radio or not reading the newspaper in India [26] are independent barriers to early breastfeeding initiation. Despite breastfeeding being of low direct cost and a highly cost-effective strategy [6, 7, 9], lack of access to information is often linked to wealth in access to services, media, and information. Secondly, three other studies with moderate quality grading [26, 28, 29] highlight lack of access of mothers in decision making as a barrier to the early initiation. Lack of mother’s involvement in decision making has been reported as reason for not initiating breastfeeding within 1 h of birth. Mothers are less likely to delay initiation if they have a final say in all categories of decision making in Nepal [28]. This was also reported in India [26] and Bangladesh [29] however was not significant after adjusting for other variables.

## Discussion

Early initiation of breastfeeding, specifically within 1 h of birth, refers to the best practice recommendation by the WHO [4]. Increasing early initiation of breastfeeding will directly support progress towards achieving MDG 4 through reduced neonatal mortality [5–7, 50] as well as through improved childhood nutrition with associations reported with reduced moderate wasting and stunting prevalence, and the incidence of acute and persistent diarrhoea in children under 5 years [51].

The findings of this systematic review suggest that achieving more widespread practice of early breastfeeding initiation hinges on multisector interventions. For instance, access to universal primary education [52] will resolve the negative impact that lack of education for mothers and fathers has on breastfeeding initiation. This is also not exclusive to South Asia as lack of education is also reported as a factor to early breastfeeding initiation in Nigeria [53], Ethiopia [54], Tanzania [55] and Malawi [56]. Similarly, promotion of gender equality and empowerment of women [52], lack of decision making power of mothers is a barrier to early initiation of breastfeeding, which is consistent with findings in Tanzania [55], and mothers-in-law are often decision makers on pregnancy and childbirth-related practices. Further, progress in maternal health and the promotion of maternal health services such as antenatal appointments, skilled birth attendance and postnatal check-up given their impact on a mother’s decision and capacity to initate breastfeeding within 1 h of delivery. This is pertinent particularly in South Asia where more than half of deliveries in several South Asian countries occur outside health facilities [57] and home delivery was identified as a barrier to early initiation of breastfeeding. This association between home delivery and delayed breastfeeding initiation is consistent with reports from Nigeria [53], Tanzania [55], Ethiopia [54] and Malawi [56]. The low use of antenatal check-up is also an observed barrier in Vietnam [58], Turkey [59], Malawi [56] and Nigeria [53]. These consistencies confirm that promoting and facilitating the use of maternal health services should be prioritised to achieve progress on early initiation of breastfeeding. Actions targeting the factors and specific barriers identified in this review will have a synergistic effect on early breastfeeding initiation and achievement of other development goals.

One of the major findings of this review is the influence of traditional beliefs and role of mother in law on breastfeeding. Traditional feeding practices, such as prelacteal feeds, misperceptions regarding colostrum, and taking advice of priests and mothers in-laws that discourage breastfeeding immediately after birth have been highlighted. Therefore, strategies that engage social and family decision-makers to shape traditional beliefs and attitudes towards safer breastfeeding practices are imperative in South Asia [60].

Policies are in place to support recommended breastfeeding practices in South Asia. With the exception of India, all South Asian countries have a national IYCF strategy officially adapted by government [61]. Similarly, all countries have a National Breastfeeding Committee, have adopted the Baby Friendly Hospital Initiative (BFHI), and implement the International Code of Marketing of Breast milk Substitutes [61]. Yet, the rates of early breastfeeding initiation in the South Asian countries remain some of the lowest in the world [1]. Filling the gap, identified in this review, in evidence concerning socio-economic and political context that influence breastfeeding practices may lead to better informed and more context-specific policies that impact more significantly. Further, the exploration of factors and barriers presented sheds light on the factors and barriers that undermine the effective implementation of policies at the individual level.

This review was influenced by several limitations thus results should not be interpreted as a necessarily definitive list of all factors and barriers experienced by women in South Asia. As the South Asia region is highly diverse and ever-changing over time, as are the situations within each country, the results of these studies based on their size and scope cannot fully represent the region as a whole. Further, as no studies from Afghanistan and Bhutan met the inclusion criteria these studies and review results may not represent those two countries. However, the inclusion dates were limited and findings presented based on countries and detailing their specify context and participant type where possible to assist the use of the findings per situation.

The nature of the evidence, the lack of strong quality studies by design and sample size, limit the overall strength of the findings however this is not a topic suited to randomised controlled trials therefore this review reflects some of the highest quality that is likely to be generated. Studies classified as ‘weak’ were retained to afford a general sense of the documented factors and barriers however interpretation of the results of those studies is taken with caution and has been noted throughout presentation of the findings. Grey literature was not included thus it is possible that relevant unpublished articles were overlooked however the lack of peer-review for grey literature inflicts quality concerns. The findings are discussed in terms of countries generally however as these are drawn from isolated and qualitative studies with some being very small sample size (six) they are not nationally representative of the factors and barriers, with the exception of eight included studies, in Bangladesh, Nepal, India and Pakistan, that analysed nationally representative surveys and are moderate in quality [25–30, 35, 43]. Nevertheless, many factors are highlighted by not only the findings of one study, but also supported by more than one study with both moderate and weak quality grading, and thus the findings can be used to design programs to increase early or timely initiation of breastfeeding and reduce neonatal deaths. Afghanistan and Bhutan rank the lowest, in D-grade, in terms of implementation of the policies and programs of the global strategy on breastfeeding [62], yet no published studies were identified concerning the factors and barriers from these two countries, highlighting an important research gap.

## Conclusion

Attention to raise rates of early breastfeeding initiation in South Asian countries is a public health priority given that the rates of early initiation of breastfeeding in the region is lowest, newborn mortality now accounts for more than half of the U5MR, and early initiation may prevent up to half of the newborn deaths and improve childhood nutritional status. This systematic review reveals that factors associated with and barriers to early initiation of breastfeeding in South Asia are predominantly on specific socioeconomic, health related and individual factors; and demand side barriers. As this study highlighted limited attention and evidence on the influence of the health care system and wider political context we suggest future studies that assess how such systems influence the early initiation of breastfeeding. Studies in Afghanistan and Bhutan would be of value to identify factors specific in these settings as this review found no studies in these countries. Further, the authors recommend national studies with sub-population representative samples providing analysis of the relative magnitude of specific factors which limit breastfeeding initiation to inform the direction of policy and resources for most effective action.

Factors and barriers manifest similarly across the region although contextual variations are observed, thus actions must be both general and aligned to specific settings. Initiatives that span the breadth of factors and directed towards local barriers are urgently needed to increase the practice of breastfeeding initiation within 1 h of birth and achieve greater reductions in neonatal mortality and improved child health in the South Asia region.

## Additional files

Additional file 1: Detailed search strategy in one database. (DOCX 26 kb) Additional file 2: Detailed characteristics of included studies. (DOC 129 kb)
