# Supplementary material for: Early initiation of breastfeeding: a systematic literature review of factors and barriers in South Asia
Source: Int Breastfeed J. 2016 Jun 18;11:17. doi: 10.1186/s13006-016-0076-7 (PMC4912741; doi:10.1186/s13006-016-0076-7)
Supplement: Additional file 1: — Detailed search strategy in one database. (DOCX 26 kb) [file 13006_2016_76_MOESM1_ESM.docx]

**Additional file 1. Full search strategy, illustration of one database**

**MEDLINE**

| **SN** | **Results** | **Search Terms and or MeSH headings** |
| --- | --- | --- |
| # 12 | 881 | #7 AND #6 AND #3  Refined by: [excluding] Publication Years=( 1976 OR 1988 OR 1979 OR 1970 OR 1986 OR 1974 OR 1989 OR 1982 OR 1975 OR 1983 OR 1977 OR 1980 OR 1963 OR 1981 OR 1966 OR 1987 OR 1967 OR 1984 OR 1968 OR 1978 OR 1972 OR 1985 ) AND Languages=( ENGLISH ) AND [excluding] Publication Types=( EDITORIAL OR COMMENT OR GUIDELINE OR LETTER OR NEWSPAPER ARTICLE )  *Databases=MEDLINE Timespan=All years* |
| # 11 | 889 | #7 AND #6 AND #3  Refined by: [excluding] Publication Years=( 1976 OR 1988 OR 1979 OR 1970 OR 1986 OR 1974 OR 1989 OR 1982 OR 1975 OR 1983 OR 1977 OR 1980 OR 1963 OR 1981 OR 1966 OR 1987 OR 1967 OR 1984 OR 1968 OR 1978 OR 1972 OR 1985 ) AND Languages=( ENGLISH )  *Databases=MEDLINE Timespan=All years* |
| # 10 | 909 | #7 AND #6 AND #3  Refined by: [excluding] Publication Years=( 1976 OR 1988 OR 1979 OR 1970 OR 1986 OR 1974 OR 1989 OR 1982 OR 1975 OR 1983 OR 1977 OR 1980 OR 1963 OR 1981 OR 1966 OR 1987 OR 1967 OR 1984 OR 1968 OR 1978 OR 1972 OR 1985 )  *Databases=MEDLINE Timespan=All years* |
| # 9 | 1108 | #7 AND #6 AND #3  *Databases=MEDLINE Timespan=All years* |
| # 8 | 4159 | #7 AND #6  *Databases=MEDLINE Timespan=All years* |
| # 7 | 538,903 | #5 OR #4  *Databases=MEDLINE Timespan=All years* |
| # 6 | 39,502 | #2 OR #1  *Databases=MEDLINE Timespan=All years* |
| # 5 | 18,008 | Topic=((((((((((((("South Asia*") OR "South East Asia*") OR "Southern Asia*") OR "South Eastern Asia*") OR "Southern East Asia*") OR SEAR) OR Afghan*) OR Bangladesh*) OR Bhutan*) OR India*) OR Maldives) OR Nepal*) OR Pakistan*) OR "Sri Lanka*")  *Databases=MEDLINE Timespan=All years* |
| # 4 | 466,089 | MeSH Heading:exp=(Asia Southeastern OR Asia)  *Databases=MEDLINE Timespan=All years* |
| # 3 | 1,644,899 | Topic=((((((initiat*) OR colostrum) OR "pre-lacteal") OR "pre lacteal") OR prelacteal) OR "early") OR delay)  *Databases=MEDLINE Timespan=All years* |
| # 2 | 39,502 | Topic=(((((((Breastfeed*) OR "Breast feed*") OR Breastfed*) OR Breast-fed) OR "Breast fed*" Breast-feed*) OR "breast milk") OR "breastmilk") OR "breast-milk")  *Databases=MEDLINE Timespan=All years* |
| # 1 | 24, 732 | MeSH Heading:exp=(breast feeding)  *Databases=MEDLINE Timespan=All years* |

Note: Last date searched 3^rd^ September, 2013
