# Supplementary material for: Early initiation of breastfeeding: a systematic literature review of factors and barriers in South Asia
Source: Int Breastfeed J. 2016 Jun 18;11:17. doi: 10.1186/s13006-016-0076-7 (PMC4912741; doi:10.1186/s13006-016-0076-7)
Supplement: Additional file 2: — Detailed characteristics of included studies. (DOC 129 kb) [file 13006_2016_76_MOESM2_ESM.doc]

**Additional file 2. Detailed characteristics of the included studies**

| **SN** | **Source** | **Study setting** | **Outcomes/ factors/barriers** | **Study Design** | **Study methods** | **Study participants** | **Sample size** | **Overall quality grading** |
| --- | --- | --- | --- | --- | --- | --- | --- | --- |
| **1** | Dibley et al. 2010 [43] | Bangladesh, India, Nepal and Sri Lanka | - Delivery by caesarean section - Home delivery - No ANC with healthcare providers - No participation in decision making - Lack of access to media | Descriptive cross sectional survey | Quantitative cross sectional study: secondary analysis of DHS (Bangladesh, Nepal and Sri Lanka) and India Family Health Survey | Women aged 15-49 years interviewed | 124, 385 ever-married women aged 15-49 years | Moderate (moderate in adjusting for confounders, and blinding; weak design) |
| **2** | Haider et al. 2010 [41] | 2 rural 1 urban area in Dhaka, Bangladesh | - Lack of knowledge - No milk secretion - Traditional belief - Mother’s ill-health - Baby’s ill-health - Grandmothers’ decision - Midwife discouraged - Lack of support | Descriptive cross sectional study | Quantitative cross-sectional followed by qualitative (42 semi structured interviews, 28 FGDs, 21 opportunistic observations and 26 Trials of Improved Practices (TIPs) | Mothers with children < 2 years. | 356 mothers with children less than 2 years of age | Weak quantitative (study design, analysis involving no statistical association, confounding not addressed);  Moderate qualitative (design, ethics not fully considered) |
| **3** | Parveen et al. 2012 [39] | Haryana, India | - Custom - Mother's illness - No milk secretion | Descriptive cross sectional study | Quantitative cross-sectional descriptive survey with SSI | Mothers with at least one child < 3 years of age | 61 mothers with at least one child under 3 years of age | Weak (confounding not addressed, weak data collection method, n=61) |
| **4** | Kaushal et al 2005 [44] | Haryana, India | - Custom | Descriptive cross sectional study quantitative | Qualitative (5 FGDs) followed by quantitative (questionnaires in 30 households) | Mothers and grandmothers with infants < 1 year. | FGD: 22 mothers, 30 grandmothers  Interview: 29 mothers, 27 grandmothers | Weak quantitative (selection, design, confounding not address);  Weak qualitative (weak data collection, analysis) |
| **5** | Badruddin et. al 1997 [37] | Karachi, Pakistan | - Mother unable to sit - Caesarean section delivery - Night time - High BP of mother | Longitudinal study | Quantitative longitudinal (cross-sectional before and after). Qualitative: FGDs and in-depth interviews | Mother-infant pairs were followed from birth to 16 weeks of age. | 102 mother-infant pairs | Weak quantitative (weak data collection, prone to bias)  Moderate qualitative (analysed, ethics not fully considered) |
| **6** | Dihidar et al. 2002 [33] | Calcutta, India | - Living in rural area | Descriptive cross sectional study | Quantitative cross sectional survey. Random sampling | Mothers, with a child < 5 years of age. | 1639 women below 45 years sub-sample analysis. (Total survey 5,161 households) | Weak (selection bias, design) |
| **7** | Senarath et al., 2012 [25] | Sri Lanka | - Male child (AOR female:0.75; 95%CI: 0.59-0.94) - LBW (AOR:2.24; 1.66-3.03) - Home delivery (AOR: 5.29; 1.66-16.88) - Caesarean section delivery (AOR: 3.30; 2.54-4.28) - Rural (AOR vs estate 0.61; 0.39-0.95) - Mother's age15-19 yrs (p<0.05) - First birth order ( p<0.05) - No previous birth - Geography: North Central (AOR: 1.96; 1.25-3.07); Sabaragamuwa province (p<0.05) | Descriptive cross sectional survey | Quantitative cross sectional: secondary analysis of DHS 2006/07 | Ever-married women 15–49 years | 2735 children under 24 months of age (Sub-sample analysis, total survey 14 692 sample size) | Moderate (moderate in adjusting confounder, and blinding; weak in design) |
| **8** | Mihrshahi et al. 2010 [29] | Bangladesh | - Education: Maternal (OR none vs secondary +: 0.61; 0.44-0.84). Husband (none vs secondary +: 0.59; 0.38-0.92) - Birth order >5 (OR: 1.76; 1.13- 2.74) - Home delivery (OR vs health facility: 0.65; 0.43-0.98) - No ANC (OR vs 3-6: 0.50; 0.4-0.73) - Mothers not watching television (OR vs watching: 0.73; 0.55-0.96); - Poorest household (AOR vs poorer: 0.61; 0.37-0.99; middle: 0.59; 0.38- 0.92, richer: 0.37; 0.23- 0.57, richest: 0.53, 0.34-0.84) - No participation in decision making (OR all categories vs none:1.60; 1.05- 2.44) - Geography (lowest for living in Barisal) | Descriptive cross sectional survey | Quantitative cross sectional: secondary analysis of DHS 2004 | Ever-married women aged 15-49 yrs, with child < 24 months of age | 2482 children under 24 months. (Sub-sample analysis, total survey 11,440 sample size) | Moderate (moderate in adjusting confounder, and blinding; weak in design) |
| **9** | Pandey et al. 2010 [28] | Nepal | - Caesarean section delivery (AOR: 4.99; 95% CI:1.27-19.52); - Delivery assistance from health professionals (OR TBA vs. professionals:0.26; 0.08-0.85) - No participation in decision making (AOR none vs 2 categories:0.47; 0.24-0.90; vs 2-4 categories: 0.63; 0.41-0.97) - Living in mountainous region (AOR vs. Terai: 0.58; 0.35-0.98) | Descriptive cross sectional survey | Quantitative cross sectional: secondary analysis of DHS 2006 | Ever-married women aged 15-49 yrs, with child < 24 months of age | 1906 children aged 0-23 months. (Sub-sample analysis, total survey 8,600 sample size) | Moderate (moderate in adjusting confounder, and blinding; weak in design) |
| **10** | Seranath et al. 2010 [35] | Sri Lanka | - Birth order 1(OR vs 2-3: 0.62; 0.42-0.93) - Caesarean section delivery (AOR: 3.23; 1.92-5.43) - ANC by midwife (AOR:1.81; 1.13-2.89) - Geography: Colombo feeder vs irrigated dry zone (OR 0.45; 0.20-0.99) | Descriptive cross sectional survey | Quantitative cross sectional: secondary analysis of DHS 2000 | Ever-married women aged 15-49 yrs, with child < 24 months of age | 1127 children aged 0-23 months. (Sub-sample analysis, total survey 6,385 sample size) | Moderate (moderate in adjusting confounders, and blinding; weak in deign) |
| **11** | Hazir et al. 2013 [27] | Pakistan | - Working mothers (p=0.001) - Caesarean section delivery (p=0.001) - Residing in Sindh Province (p=0.001) | Descriptive cross sectional survey | Quantitative cross sectional: secondary analysis of DHS 2006/07 | Ever-married women, with child < 24 months of age | 3103 children aged 0-23 months. (Sub-sample analysis, total survey 10,023 sample size) | Moderate (moderate in adjusting confounders, and blinding; weak in design) |
| **12** | Khadduri et al. 2008 [47] | Haripur district, Pakistan | - Traditional mixture for prelacteal feeding | Descriptive cross sectional study | Qualitative: 43 semi-structured interviews, 34 FGDs | Health workers, men, women aged 15-49 with a child born in the past 6 months, untrained TBAs. | 64 interviews, 34 FGDs | Moderate qualitative |
| **13** | Bandyopadhyay et al. 2009 [48] | Rural Bengal, India | - Perception that first milk is harmful to the baby; insufficient milk; perception that milk comes only after 48 hours | Descriptive cross sectional study | Quantitative data cross sectional. Qualitative: 30 in depth interviews, 12 case studies | Women, married aged 13-49 with one+ living child. Case studies: women of various castes | 402 women for quantitative survey  30 women for In-depth interview | Weak quantitative (design, confounders not addressed)  Moderate qualitative |
| **14** | Patel et al. 2010 [26] | India | - No education (AOR vs secondary +: 0.79; 95% CI: 0.66-0.94) - Mothers aged 15-19 years (AOR vs 20−34: 0.80; 0.65-0.98) - No education of husband (AOR vs primary:0.72; 0,62- 0.84; vs secondary +: 0.63, 0.51-0.79) - Home delivery (AOR vs health facility 0.78; 0.65-0.93) - Caesarean section delivery (AOR: 2.52; 1.97-3.22) - No ANC (AOR vs 1−2 visits: 0.62; 0.50-0.79; vs 3−6 visits: 0.50; 0.40-0.63; vs ≥ 7 visits 0.48; 0.35-0.64, p<0.001)   Bivariate analysis   - No PNC (OR: 2.2; 1.93-2.52, p<0.001) - Lowest wealth quintile: OR vs middle: 0.65; 0.53-0.79; richer: 0.59; 0.48-0.73) - No participation in decision making (OR vs 3−4 categories:0.78;0.64-0.93; vs 5−6 categories: 0.65; 0.54-0.77) - Not listening to radio (OR vs listening: 0.76;0.66-0.87) - Not reading newspaper (OR vs reading: 0.55; 0.48- 0.62) - Not watching television (OR vs watching: 0.59; 0.51-0.68) - Rural (OR vs urban:0.72; 0.61- 0.86) - Living in Central geographical region (AOR:1.78; 1.39-2.28) | Descriptive cross sectional study | Quantitative cross sectional: secondary analysis of National Family Health Survey 2005-06 | Ever-married women aged 15-49, with child <24 months of age | 20,108 children <24 months of age. (Sub-sample analysis, total survey 124,385 sample size) | Moderate (moderate in adjusting confounders, and blinding; weak in design) |
| **15** | Subedi et al. 2012 [31] | Chepang community, Nepal | - Illiterate - No ANC - Home delivery | Descriptive cross sectional survey | Quantitative cross-sectional survey | Mothers of Chepang ethnicity, with child <2 years of age | 360 mothers | Weak (design, data collection methods) |
| **16** | Subba et al. 2007 [34] | Pokhara, Nepal | - Nuclear family (p=0.02) - Smaller family size (p=0.02) | Descriptive cross sectional survey | Quantitative cross-sectional: hospital based survey | Mothers - attendees of immunization clinic | 168 mothers | Weak (weak design, confounders not addressed) |
| **17** | Abdulraheem and Binns 2007 [42] | Maldives (several islands) | - Caesarean section delivery less likely to early initiate within one hour | Descriptive cross sectional survey | Quantitative cross-sectional survey | Mothers | 251 mothers | Weak (weak design, confounders not addressed) |
| **18** | Athavale et al. 2004 [36] | Urban Health Centre, Nagpur, India | - Caesarean section delivery (x^2^= 26.08, p 0.001) - Prelacteal feeding (x^2^= 23.27, p<0.001) - Discarding colostrum ( x^2^= 13.21, p<0.01) - Premature baby ( x^2^= 17.03, p<0.001) - Parity 1(x^2^= 13.05, p<0.01) | Descriptive cross sectional survey | Quantitative cross-sectional survey | Mothers with infants attending outpatient department of Urban Government Health Centre | 200 mothers | Weak (weak design, confounders not addressed) |
| **19** | Ekambaram et al. 2010 [38] | Tertiary care hospital, South India | - Newborn was ill (34%) - Delay in shifting from labour room (25%) - Mother's too tired, unconscious (14%) - Baby was sleeping (5%), | Descriptive cross sectional survey | Quantitative cross-sectional survey | Post natal mothers | 100 mothers | Weak (weak design, data collection method, confounders not addressed) |
| **20** | Moran et al. 2009 [49] | Slum residents Dhaka, Bangladesh | - Perception of milk insufficiency | Descriptive cross sectional study | Quantitative cross-sectional survey. Qualitative: 36 in-depth semi-structured interviews | Survey: Women recently given birth. Interviews: pregnant women, women had at least 1 birth | 1256 women for survey; and 18 pregnant women for qualitative interview | Weak quantitative (weak design, weak data collection)  Moderate qualitative design (weak recruitment strategy) |
| **21** | Fikree et al. 2005 [46] | Karachi, Pakistan | - Traditional feeding practices and perceived health benefits of delayed initiation | Descriptive cross sectional study | Quantitative cross sectional survey. Qualitative: 5 FGDs, 15 semi-structured interviews. | Survey: Women recently given birth. Qualitative: women, TBAs | Survey: 525 women  FGD;s 5 FGDs , 8-10 participants each; 15 in-depth interviews | Moderate quantitative (weak design)  Moderate qualitative (recruitment strategy, ethics not considered) |
| **22** | Rahman et al. 2011 [30] | Bangladesh | - No ANC - Poorest wealth quintile - Delivery assistance by non-medically trained provider - No Education | Descriptive cross sectional survey | Quantitative cross sectional: secondary analysis of DHS 2007 | Ever-married women aged 15–19 with non-institutional birth, and a child <3 years | 580 women | Moderate (moderate in adjusting confounder, and blinding; strong in terms of removing selection bias weak in design) |
| **23** | Ali et al. 2011 [32] | Semi urban community in Pakistan | - Lack of education (p<0.05) | Descriptive cross sectional survey | Quantitative cross-sectional survey | Mothers, delivered a single, full-term, healthy birth weight. | 200 mothers | Weak (weak design, confounders not addressed) |
| **24** | Digra et al. 2012 [45] | Jammu, India | - Self-decision (22.2%) - Advice of priest (35%) - Advice of elderly lady in family (20.4%) | Descriptive cross sectional survey | Quantitative cross-sectional survey | Mothers, aged 20-40 years | 500 mothers | Weak (weak design, data collection method, confounders not addressed) |
| **25** | Premani et al. 2011[40] | Karachi, Pakistan | - Mothers too tired after delivery | Descriptive cross sectional study | Qualitative: interviews | Women aged 20-35 years, currently or discontinued breastfeeding | 6 women | Weak (weak design, recruitment strategy, ethical issues not fully considered) |
